# The Application and Limitation of Universal Chloroplast Markers in Discriminating East Asian Evergreen Oaks

**DOI:** 10.3389/fpls.2018.00569

**Published:** 2018-05-08

**Authors:** Mengxiao Yan, Yanshi Xiong, Ruibin Liu, Min Deng, Jiaojiao Song

**Affiliations:** ^1^Shanghai Chenshan Plant Science Research Center, Chinese Academy of Sciences, Shanghai Chenshan Botanical Garden, Shanghai, China; ^2^College of Life and Environmental Sciences, Shanghai Normal University, Shanghai, China; ^3^College of Life Sciences, Shangrao Normal University, Shangrao, China

**Keywords:** *cp*DNA, DNA barcoding, genetic diversity, *Quercus*, section *Cyclobalanopsis*, section *Ilex*

## Abstract

The East Asian subtropics mostly occupied by evergreen broad-leaved forests (EBLFs), is one of the global diversity centers for evergreen oaks. Evergreen oaks are keystone canopy trees in EBLFs with important ecosystem function and crucial significance for regional biodiversity conservation. However, the species composition and diversity of Asian evergreen oaks are poorly understood. Here, we test whether the four chloroplast markers *atp*I-*at*pH, *mat*K, *psb*A-*trn*H, and *ycf*1, can discriminate the two evergreen oak sections in Asia – *Cyclobalanopsis* and *Ilex.* Two hundred and seventy-two individuals representing 57 species were scanned and 17 species from other oaks sections were included for phylogenetic reconstruction. The genetic diversity of the *Quercus* sections was also compared. Overall, we found that universal chloroplast DNA (*cp*DNA) barcoding markers could resolve two clades in *Quercus*, i.e., subgenus *Cerris* (Old World Clade) and subgenus *Quercus* (New World Clade). The chloroplast markers distinguished the main sections, with few exceptions. Each *cp*DNA region showed no barcoding gap and none of them provided good resolution at the species level. The best species resolution (27.78%) was obtained when three or four markers were combined and analyzed using BLAST. The high conservation of the *cp*DNA and complicated evolutionary patterns, due to incomplete lineage sorting, interspecific hybridization and introgressions may hinder the ability of *cp*DNA markers to discriminate different species. When comparing diversification pattern across *Quercus* sections (*Cyclobalanopsis, Ilex, Cerris, Quercus*, and *Protobalanus*), we found that section *Ilex* was the most genetically diverse, and section *Cyclobalanopsis* was lower genetically diverse. This diversification pattern may have resulted from the interplay of the Eurasia Cenozoic tectonic movements, climate changes and different niches of their ancestral lineages.

## Introduction

Understanding the biodiversity of ecosystems is critical for revealing the biome assembly and its function in different ecosystems (Hebert et al., 2003; Lahaye et al., 2008; Pitman et al., 2008). Taxonomy based on morphological features has enriched our understanding of biodiversity for over 300 years, and provide an inventory of global biodiversity. However, the morphology based taxonomy system has shortcomings (Hebert et al., 2003; Vilgalys, 2003; Gonzalez et al., 2009; Goldstein and DeSalle, 2011). For example, the key diagnostic traits may be under selection or subjected to parallel evolution providing false information on the identity of the taxa. Complementing techniques such as DNA sequences under neutral evolution have become an efficient way to identify species (Hollingsworth, 2007; CBOL Plant Working Group, 2009; Valentini et al., 2009; Hollingsworth et al., 2016). DNA barcoding has become instrumental in plant research for identification of cryptic species (Bickford et al., 2007; Miwa et al., 2009; Liu et al., 2011), biodiversity assessments (Lahaye et al., 2008; Gonzalez et al., 2009; von Cräutlein et al., 2011; Yan et al., 2015), community phylogeny (Kress et al., 2009, 2010), conservation biology (Laiou et al., 2013; Veldman et al., 2014; Liu et al., 2015; Shapcott et al., 2015), invasive biology (Armstrong and Ball, 2005; Bleeker et al., 2008; Newmaster and Ragupathy, 2009; Van De Wiel et al., 2009), and disease and pest management (Ball and Armstrong, 2006; Lee et al., 2012; Montecchio and Faccoli, 2014; Shapcott et al., 2015). At present, the resolution of several candidate DNA barcodes has been tested in many plant groups (Clerc-Blain et al., 2010; Gao et al., 2010; Liu et al., 2011; Yu et al., 2011; Wang D.Y. et al., 2017). However, there is a challenge for DNA barcoding to discriminate closely related species, especially for those with shallow phylogeny and complex evolutionary history (Newmaster et al., 2008; Chen et al., 2015; Yan et al., 2015), such as species-rich genera, *Quercus*.

The genus *Quercus* (Fagaceae) has ca. 400–500 species, which are widely distributed in the warm temperate forests of the Northern Hemisphere (Camus, 1936–1954; Nixon, 1993). Many species of the genus are the canopy or dominant trees in regional EBLFs and play an important role in the function and service of ecosystem (Fang and Yoda, 1991; Nixon, 2006; Petit et al., 2013). The species identification and taxonomy in oaks are notoriously difficult (Manos et al., 1999; Simeone et al., 2013; Hubert et al., 2014), especially for Asian evergreen oaks that are composed of two species-rich sections (*Cyclobalanopsis* and *Ilex*) (Denk and Grimm, 2010; Denk et al., 2017). Species of section *Cyclobalanopsis* are the dominant trees in Asian (sub)tropical EBLFs with ca. 90–120 species (Denk and Grimm, 2010; Denk et al., 2017). Species of section *Ilex* are widely distributed in Eurasian low-middle latitude habitats comprising ca. 35–36 species (Denk and Grimm, 2010; Simeone et al., 2016). Unfortunately, the wide range of EBLFs in East Asia is now greatly diminished as a result of intensified human activities (Cao and Zhang, 1997; Tang, 2010). At least 1/3 oak species in China are now endangered or threatened due to the severe habitats loss and human activities (Deng et al., 2013a). Therefore, understanding the species composition of East Asian evergreen oak forests is important for future conservation efforts. Although the Flora of China and other regional floristic works had been finished for decades (e.g., Huang et al., 1999; Phengklai et al., 2008), the oak taxonomy and their systematic placement are still not well resolved, in part due to the high level of intra- and inter-species genetic variation. Even the fine anatomy features of the East Asian evergreen oaks, such as leaf epidermal features (Deng et al., 2014, 2017b), pollen morphology (Denk and Grimm, 2009; Deng et al., 2013b; Denk and Tekleva, 2014), and wood anatomy (Zhao et al., 2007) had been comprehensively studied in recent years. But these works do not provide useful diagnostic features to clarify species identity, and suggest paraphyletic evolution of taxonomical traits. Moreover, the interspecies gene flow has blunted genetic integrity and reduced the ability to distinguish some East Asian evergreen oaks (Tamaki and Okada, 2014; An et al., 2017).

Chloroplast DNA sequences have been applied for phylogenetic analysis and DNA barcoding of species of section *Ilex* in the Mediterranean (Piredda et al., 2011; Simeone et al., 2013) and China (Yang et al., 2017), providing insights into the evolutionary history of oak taxa. However, these studies included limited taxa and geographic regions (Europe or small region of China), therefore did not provide enough information on DNA barcoding for evergreen oaks. For the species-rich section *Cyclobalanopsis* in East Asia, little is known about evolution of chloroplast genome and phylogeny. So far, only the intergenic region of the chloroplast *trn*T-*trn*L was applied to distinguish six species of section *Cyclobalanopsis* distributed in Japan, and proved to have moderate efficiency (Ohyama et al., 2001). Recent studies using *cp*DNA show that the species of section *Cyclobalanopsis*, e.g., *Q. glauca* (Xu et al., 2015), *Q. schottkyana* (Jiang et al., 2016), *Q. arbutifolia* (Xu et al., 2016), and *Q. kerrii* (Jiang et al., 2018), contain high variable regions to infer population history, but the resolution of *cp*DNA markers in discriminating section *Cyclobalanopsis* species is still unknown.

Evergreen broad-leaved forests potentially cover a wide zone of monsoon-dominated regions in East Asia (Fang and Yoda, 1991). This region, as a global biodiversity hotspot, has the world’s richest flora harboring remarkable array of endemic, relic and endangered species (Myers et al., 2000; Yang et al., 2004; López-Pujol et al., 2006), especially with many endemic and endangered oak species (Menitsky, 1984; Luo and Zhou, 2000; Deng et al., 2013a). Effective DNA barcodes for species identification, conservation and resource utilization are extremely necessary in East Asian evergreen oaks. A further DNA barcoding study with comprehensive sampling on species population from the main distribution ranges is needed. In addition, comparing of the genetic diversity patterns across the main lineages in *Quercus* can provide more information to infer the driving forces which might contribute to their divergence. Furthermore, the knowledge on biodiversity inventory in East Asian subtropics is far from enough, and new species are continuously being identified in recent years (e.g., Xiao et al., 2015; Liang et al., 2016; Jiang et al., 2017; Wang C.W. et al., 2017). DNA barcoding of keystone species in this region can provide crucial insight into the mechanism of plant community assembly and evolutionary trajectory of the East Asian regional biota.

In this study, we comprehensively collected East Asian evergreen oaks aiming to address the following issues: (1) to reveal the discrimination ability of *cp*DNA barcode markers in East Asian evergreen oaks; (2) to compare the genetic diversity of the main sections in *Quercus*; (3) to explore the possible applications of *cp*DNA markers into the taxonomic and phylogenetic approaches. Our study provides insights into the species identity of Asian oaks and important information for biogeographic and population genetics of these unique oaks.

## Materials and Methods

### Ethics Statement

Sampling of oak species and other Fagaceae plants were granted and supported by National Forestry Bureau of China and Local National Nature Reserves.

### Plant Materials

One hundred and forty-seven individuals belonging to 29 species of *Quercus* section *Cyclobalanopsis* from East Asia and 125 individuals of the 28 species of section *Ilex* from East Asia were included in our studies. The sampling range covered the key distribution range of East Asian evergreen oaks in China, Japan, Vietnam, and Nepal. Additionally, individuals of five species of section *Ilex* from the Mediterranean were analyzed. The final dataset also included four species of section *Cerris* (18 individuals, from Eurasia), seven species of section *Quercus* (11 individuals, from North America and Eurasia), five species of the American section *Lobatae* (7 individuals), and one species (1 individual) of section *Protobalanus* from western North America. One individual of *Lithocarpus henryi* was used as outgroup to root the tree of genus *Quercus*. Sequences of eight species (16 individuals) were downloaded from GenBank. Detailed information of samples used in this study is listed in Supplementary Table S1.

Healthy leaves of each individual were collected and dried instantly in silica gel for DNA extraction. Voucher specimens of each individual were deposited in the Herbarium of the Shanghai Chenshan Botanical Garden (CSH).

### DNA Extraction, PCR, and Sequencing Protocols

Genomic DNA was extracted using the modified CTAB method (Doyle and Doyle, 1987). Four *cp*DNA regions, the *psb*A-*trn*H intergenic spacer, a part of the *mat*K gene, the *atp*I-*at*pH intergenic spacer and a portion of the *ycf*1 region were amplified using PCR and bidirectionally sequenced for analyses. PCR reactions were performed according to previously described method (Xu et al., 2015). The primer sequences used to amplify these regions are summarized in Supplementary Table S2. The sequences obtained in this study have been uploaded to GenBank (accession numbers: MH058100-MH059477).

Sequencher 4.01 (Gene Codes Corp., Ann Arbor, MI, United States) was used to assemble and edit sequences. The DNA sequences were aligned using the package Muscle (Edgar, 2004) implemented in MEGA 7.0.21^1^ (Kumar et al., 2016) with subsequent manual adjustment.

### DNA Barcoding Analyses

DNA polymorphisms were examined using DnaSP 5.10 (Librado and Rozas, 2009). To evaluate species discrimination success, four widely used methods, genetic distance-based, similarity-based, tree-based and diagnostic method, were applied to the four *cp*DNA markers and all their possible combinations.

Genetic distance-based method: *p*-distances of the four plastid regions were calculated in MEGA 7.0.21 (Kumar et al., 2016) (see footnote 1). We measured three parameters to determine intraspecific variation (Meyer and Paulay, 2005; Lahaye et al., 2008). First, we calculated average intraspecific difference between all samples collected. Second, we measured theta (𝜃), which means the average *p*-distance within each species. Theta eliminates biases resulted by unequal sampling between species. Lastly, we calculated average coalescent depth, which is the maximum intraspecific distance within each species. Average interspecific distance and smallest interspecific distance were used to characterize interspecific divergence (Meier et al., 2008). The distribution of intraspecific versus interspecific variability was compared using DNA barcoding gaps. Differentiations between the intraspecific and interspecific *p*-distance for each candidate barcode were also compared using the Wilcoxon signed rank tests in R 3.2.3 (R Core Team, 2015). To explore potential species groups, the obtained *p*-distance matrices for each barcode candidate and all combinations were analyzed using the Automatic Barcode Gap Discovery (ABGD) under default setting (Puillandre et al., 2012). If conspecific individuals were partitioned into the same group without sequences from other species, the species was considered as successfully delimited.

Similarity-based method: Sequences of the four candidate barcodes and all possible combinations were built as 15 local reference databases using NCBI-blast-2.6.0+ (Camacho et al., 2009; Tao, 2010). Each barcode sequence was then queried using the blastn command against its local reference database. When all individuals of a species had a top hit to conspecific individuals, species discrimination was considered successful.

Tree-based method: Bayesian trees were constructed using MrBayes 3.2.6 (Ronquist et al., 2012). Model of substitution was selected by Modeltest 3.7 (Posada and Crandall, 1998) based on the akaike information criterion (AIC). Two parallel Markov Chain Monte Carlo (MCMC) runs were performed for 20 million generations. The trees were sampled every 1,000 generations and inspected in Tracer v1.6^2^. The first 15% trees were discarded as burn-in. Species discrimination was considered as successful only when all the conspecific individuals formed a monophyletic clade.

Diagnostic method: BLOG 2.0 (Barcoding with LOGic) was used for species identification under a Logic Mining method, which identifies the species in terms of location of key diagnostic nucleotides from the training sequences to classify species (Weitschek et al., 2013). Each candidate barcode and combination were tested with a single file input, with 80% of the sequences as the training data, and 20% as the test data. A species was considered as successfully delimited if conspecific individuals were correctly identified in both training and test datasets.

### Phylogeographical and Genetic Diversity Analysis

Haplotypes were extracted by DnaSP 5.10 (Librado and Rozas, 2009). To measure the level of genetic variation, variable sites, average pairwise differences per base pair between sequence (nucleotide diversity) (Nei and Li, 1979) and haplotype diversity (Hd) were calculated using DnaSP 5.10 (Librado and Rozas, 2009). The genetic distances (*p*-distances) of each section were calculated in MEGA 7.0.21 (Kumar et al., 2016) (see footnote 1) for further comparison of genetic diversity. A principal coordinate analysis (PCoA) was performed using GenAlEx 6.5 (Peakall and Smouse, 2012) to illustrate both the similarity between different sections and the genetic diversity of each section.

## Results

### PCR Success and Sequence Characteristics

The four chloroplast candidate barcodes showed high amplification success rates (100%) at the species level. At individual level, *mat*K, *psb*A-*trn*H, and *ycf*1 had high success rates (92.2–100%) (**Table 1**), while the *atp*I-*at*pH had the lowest success rate, potentially due to the long PCR product that was being amplified.

**Table 1:** Characteristics of the four *cp*DNA markers used in this study.

					Vs					
Barcode	*N*	Ns	PCR	L	Number	%	Indel	Pi	Hn	Hd
*atp*I-*atp*H	345	72	92.2	1,187	128	10.78	99	0.00448	77	0.851
*mat*K	374	74	100	691	64	9.26	3	0.00467	64	0.819
*psb*A-*trn*H	357	74	95.5	554	64	11.55	26	0.02012	68	0.905
*ycf*1	363	74	97.1	796	90	11.31	43	0.00954	66	0.827

*N, number of samples; Ns, number of species; PCR success (%); L, aligned length; Vs, variable sites; Pi, nucleotide diversity; Hn, number of haplotypes; Hd, haplotype diversity.*

A total of 1,439 sequences were available for further analysis, of which 1,375 were newly generated in this study. Among the four *cp*DNA markers, the aligned lengths ranged from 554 bp for the *mat*K to 1,187 bp for the *atp*I-*atp*H. The *atp*I-*atp*H region showed the highest number of variable sites, while the *mat*K and *psb*A-*trn*H had the lowest variable sites (**Table 1**). The *atp*I-*atp*H region also had the highest number of indels (99), followed by *ycf*1 (43), *psb*A-*trn*H (26) and *mat*K (3). Among the four chloroplast loci, *psb*A-*trn*H was the highest haplotype diversity, followed by *atp*I-*atp*H, *ycf*1 and *mat*K (in descending order of Hd).

### Genetic Divergence Within and Between Species

*Psb*A-*trn*H exhibited the highest level of intra-species variation, followed by *ycf*1, *atp*I-*atp*H and *mat*K. When comparing the interspecific genetic divergence among the four candidate barcodes, *psb*A-*trn*H region exhibited the highest interspecific divergence, followed by *ycf*1, *atp*I-*atp*H, and *mat*K (**Table 1**). This analysis demonstrated that *psb*A-*trn*H sequences provide the most suitable DNA barcodes.

### DNA Barcoding Gap Assessment

**FIGURE 1:**
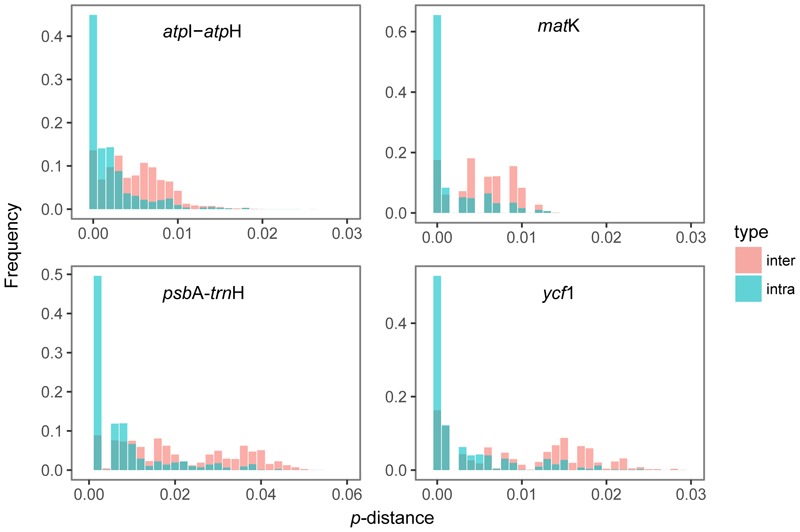
Relative distribution of intra- and inter-specific divergence of the four *cp*DNA markers.

The distribution of intraspecific and interspecific variation of four single markers lacked a distinct gap. Among the single locus, *psb*A-*trn*H had the highest variation between the distribution range of interspecific and intraspecific distances (**Figure 1**). Wilcoxon rank sum tests show that the intraspecific distances were always significantly lower than the interspecific distances (**Table 2**). However, the maximum intraspecific distances of four loci were higher than the minimum interspecific distance (**Table 2**).

**Table 2:** Comparison of inter- and intraspecific *p*-distance of the four chloroplast markers and Wilcoxon rank sum test between inter- and intraspecific *p*-distance.

Barcode	Intraspecific distance (mean)	Theta (mean)	Coalescent depth (mean)	Interspecific distance (mean)	Wilcoxon rank sum test	
					W statistic	*p*-value
*atp*I-*atp*H	0–0.0180 (0.0020)	0–0.0097 (0.0023)	0–0.0180 (0.0046)	0–0.0260 (0.0048)	15488000	<0.001
*mat*K	0–0.0130 (0.0017)	0–0.0100 (0.0022)	0–0.0130 (0.0039)	0–0.0140 (0.0052)	20437000	<0.001
*psb*A-*trn*H	0–0.0430 (0.0069)	0–0.0380 (0.0082)	0–0.0430 (0.0145)	0–0.0530 (0.0201)	14029000	<0.001
*ycf*1	0–0.0240 (0.0030)	0–0.0153 (0.0036)	0–0.0240 (0.0072)	0–0.0300 (0.0095)	18544000	<0.001

### Species Identification Efficiency of Chloroplast Loci

**FIGURE 2:**
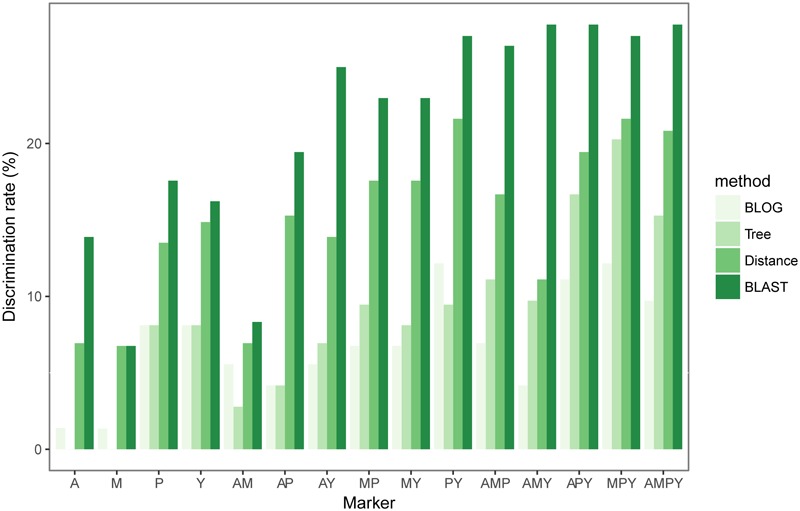
Species resolution of the four *cp*DNA markers and all combinations based on different methods.

Rates of species resolution depended on the analytic methods used (**Figure 2** and Supplementary Table S3). Overall, BLAST provided the highest species discrimination rates (6.76–27.78%) among single marker or combined markers, followed by distance-based method (6.76–21.62%) and tree-based (0–20.27%) method. BLOG provided the lowest species resolution (1.35–12.16%) overall (**Figure 2** and Supplementary Table S3). The diagnostic nucleotide of the highest species identification identified by BLOG is listed in Supplementary Table S4.

Among the four single markers, *psb*A-*trn*H region showed the highest species discrimination rate for all methods except the distance-based method. When distance-based method was used, *ycf*1 exhibited the highest species discrimination rate (**Figure 2** and Supplementary Table S3). In general, combinations with more loci could increase the probability that a species is identified correctly. Using three different combinations (*atp*I-*atp*H + *mat*K + *psb*A-*trn*H, *atp*I-*atp*H + *mat*K + *ycf*1 and *atp*I-*atp*H + *mat*K + *psb*A-*trn*H + *ycf*1) with BLAST, they all generated the highest species identification rate (27.78%) (**Figure 2** and Supplementary Table S3).

### Phylogenetic Relationship

**FIGURE 3:**
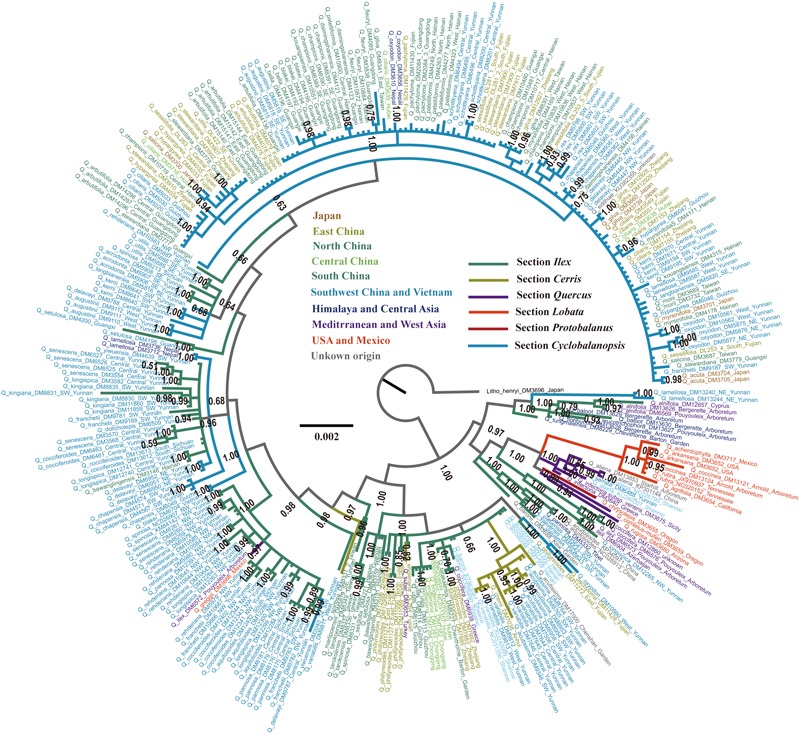
BI phylogram of *Quercus* inferred using combined *cp*DNA sequences. The tree was rooted using *Lithocarpus henryi* as the outgroup. Numbers close to the nodes are posterior probability values. Branch colors indicate different sections and colors of individuals indicate their localities with detail indication in the center of this figure.

Subgenus *Quercus*, also known as the New World Clade that includes sections *Lobatae, Protobalanus*, and *Quercus*, formed a monophyletic clade (posterior probability = 1.00) (**Figure 3**). Section *Lobatae* was the only monophyletic clade (posterior probability = 1.00) inferred in this study. Two lineages were resolved in section *Quercus*, one with Eurasian species and the other one with the two American species (**Figure 3**). Section *Protobalanus* (*Q. chrysolepis*) was the sister group to the American lineage of section *Quercus* (**Figure 3**). However, we did not find strong support for the monophyletic status of subgenus *Cerris* (the Old World Clade). Species in section *Cerris* almost formed an monophyletic lineage, except for two individuals. The Mediterranean *Q. suber* mixed with species of section *Ilex* and an individual of East Asian *Q. variabilis* nested in a sister lineage to a section *Ilex*-*Cyclobalanopsis* mixed lineage (**Figure 3**). We also inferred one main clade and 11 small clades in section *Cyclobalanopsis*. However, we did not find strong support for the monophyletic status of section *Cyclobalanopsis* (**Figure 3**). Species within the 11 small *Cyclobalanopsis* clades were mixed with section *Ilex* species. In turn, the *cp*DNA based phylogenetic reconstruction inferred that section *Ilex* was polyphyletic and its evolutionary history appeared rather complicated compared to the other sections in *Quercus* (**Figure 3**).

**FIGURE 4:**
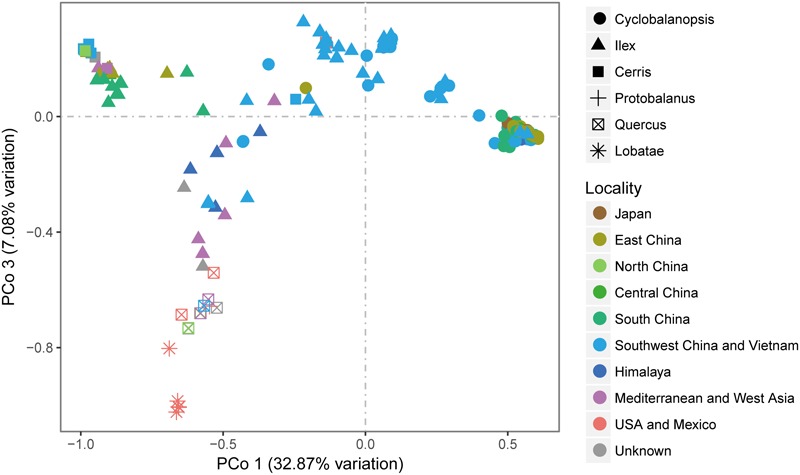
PCoA analysis of *Quercus* species based on the genetic distance obtained from combined *cp*DNA sequences.

Similar to the phylogenetic reconstruction results, PCoA using genetic distance among all the six sections (**Figure 4**) showed that section *Cerris* is closely to a group of East Asian subtropical species, including *Q. acrodonta, Q. baronii, Q. phillyreoides, Q. dolicholepis*, and *Q. engleriana*. Species of section *Quercus* from Oregon showed close similarity to section *Lobatae.* The major clade of section *Cyclobalanopsis* was relatively isolated, with only a few samples from Yunnan, SW China, are closely related to the individuals of section *Ilex* from the same region.

### Genetic Diversity Pattern

**FIGURE 5:**
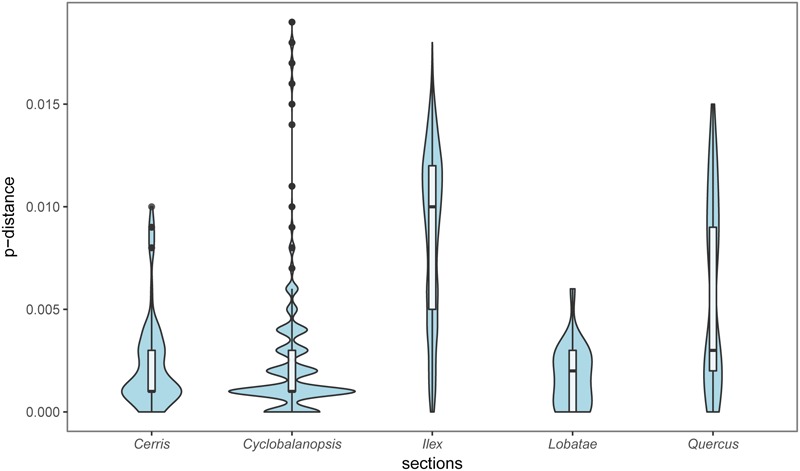
Genetic divergence of the five sections of genus *Quercus* based on the combined *cp*DNA sequences.

Section *Ilex* showed the highest diversity values for all the parameters (**Table 3** and **Figure 5**), and formed three clusters in different quadrants on the PCoA plot (**Figure 4**). This suggests that section *Ilex* was the most diverse section in *Quercus*. Section *Quercus* was the second diverse section, with the second highest *p*-distance and haplotype diversity even only having a small number of samples (**Table 3** and **Figure 5**). However, section *Cyclobalanopsis* with both high number of species and population sampling, yielded low genetic diversity levels, as the *p*-distance concentrated on low values (**Figure 5**) and the majority of individuals formed a small cluster in the PCoA analysis (**Figure 5**). Sections *Cerris* and *Lobatae* exhibited low genetic diversity as well (**Table 3** and **Figure 5**). Nonetheless, we could not rule out the possibility that the low diversity might be caused by limited sampling, as we only covered four species and 18 individuals of section *Cerris* and five species (seven individuals) of section *Lobatae*.

**Table 3:** Genetic diversity obtained from the combined *cp*DNA sequences.

Dataset (section)	*N*	Ns	*p*-distance	Vs		Pi	Hn	Hd	Indel
				Number	%				
*Cyclobalanopsis*	147	29	0–0.019 (0.0020 ± 0.0031)	134	4.19	0.00205	54	0.837	55
*Ilex*	125	28	0–0.018 (0.0090 ± 0.0041)	220	6.88	0.00867	77	0.989	70
*Cerris*	18	4	0–0.011 (0.0025 ± 0.0026)	51	1.59	0.00257	9	0.895	32
*Quercus*	11	7	0–0.013 (0.0059 ± 0.0045)	67	2.10	0.00588	9	0.964	25
*Lobatae*	7	5	0–0.006 (0.0017 ± 0.0015)	17	0.53	0.00867	4	0.714	4
*Protobalanus*	1	1	–	–	–	–	–	–	–

*N, number of samples; Ns, number of species; *p*-distance [min. – max. (mean ± SD)]; Vs, variable sites; Pi, nucleotide diversity; Hn, number of haplotypes; Hd, haplotype diversity.*

## Discussion

### The Application of *cp*DNA Markers on Oak Taxonomy and Systematics

We were able to successfully amplify and sequence the four *cp*DNA markers at high success rates (over 90%). There were differences in discrimination ability among each *cp*DNA marker. *Psb*A-*trn*H and *ycf*1 had the highest discrimination ability whereas *mat*K had the lowest. The chloroplast region *psb*A-*trn*H had the highest discriminatory power in other plant groups and community phylogeny (Kress et al., 2010; Hollingsworth et al., 2011). Three different combinations (*atp*I-*atp*H + *mat*K + *psb*A-*trn*H, *atp*I-*atp*H + *mat*K + *ycf*1 and *atp*I-*atp*H + *mat*K + *psb*A-*trn*H + *ycf*1) generated the highest species identification rate (27.78%) when used BLAST. Unfortunately, the overall identification success rates are not ideal and even the highest species resolution was below 30%. Meanwhile, none of the four chloroplast regions showed a barcode gap, which is an essential evaluation index for DNA barcodes (Hebert et al., 2003; Meier et al., 2006, 2008). The poor species resolution of universal chloroplast markers has been reported in East Asian oaks (Ohyama et al., 2001; Dong et al., 2015; Yang et al., 2017) and Euro-Mediterranean oaks (Piredda et al., 2011; Simeone et al., 2013), suggesting that these *cp*DNAs may not be ideal for resolving species identity in closely related oaks.

However, the universal *cp*DNA markers can provide information on resolving main infrageneric groups in oaks. First, two large clades of oaks, subgenera *Cerris* and *Quercus* could be distinguished when using the concatenated marker. Second, section *Cerris* is almost recognizable among the East Asian oaks, because it formed a monophyletic lineage with only two exceptional individuals falling into different groups. Third, most individuals of section *Cyclobalanopsi*s (84.4%) formed a large lineage that was easily distinguished from section *Ilex*. Few individuals of section *Ilex* showed close relationship with few species in section *Cyclobalanopsis* from SW China. In addition to the concentric rings and scales on the cupule wall, section *Cyclobalanopsis* and *Ilex* have distinct morphological traits, such as leaf architecture structures (Zhou et al., 1995; Luo and Zhou, 2002), leaf blade shape and margin teeth (Menitsky, 1984; Huang et al., 1999) that can easily be used to distinguish the two sections.

Chloroplast markers are effective barcodes in discriminating many plant species, such as Myristicaceae (Newmaster et al., 2008), *Pedicularis* (Yu et al., 2011), Asteraceae (Gao et al., 2010), *Carex* and *Kobresia* (Clerc-Blain et al., 2010). However, they have limitations when discriminating closely related species, which is quite common, not only in oaks but also in other plant taxa, such as *Salix* (Percy et al., 2014), *Rhododendron* (Yan et al., 2015), *Viburnum* (Clement and Donoghue, 2012), and *Curcuma* (Chen et al., 2015). In this study, various *cp*DNA haplotypes exist within a species and some of these haplotypes are shared interspecifically. Recent phylogeographic studies of the section *Ilex* from the Himalayas (Feng et al., 2016; Meng et al., 2017) and the pan-Mediterranean (Simeone et al., 2016; Vitelli et al., 2017) also showed similar scenario. Moreover, even using the complete plastid genome sequences for phylogenetic reconstruction, the monophyletic status of species was still rare in section *Quercus* (Pham et al., 2017). The chloroplast genome may not be an efficient marker for fine-scale DNA barcoding nor for inferring systematics of closely related oak species (Pham et al., 2017). Therefore, either subsets of chloroplast barcode sequences, or the complete chloroplast genome are unlikely to provide confident identification of the source species. All these facts restrict the application of *cp*DNA sequences on species identification in closely related oaks.

Aside from *cp*DNA markers, ITS (incl. ITS1, 5.8S, ITS2) has been used as a universal DNA barcode marker for species-level phylogenetic studies (Kress et al., 2005; Hughes et al., 2006). The *nr*DNA ITS and 5S-IGS have been applied to infer oak phylogeny, which roughly illustrate the phylogeny skeleton of the main lineages in *Quercus*, however, the deep node phylogeny among sections are not resolved (Manos et al., 1999; Denk and Grimm, 2010). Indeed, orthologous sequences of *nr*DNA has better resolution than *cp*DNA at low taxonomical level to infer the phylogeny of oaks due to its biparental inheritance (Simeone et al., 2013). Nonetheless, *nr*DNA in oaks has paralogous copies and pseudogenes (Mayol and Rosselló, 2001; Bellarosa et al., 2005; Ma, 2006). Incorporating ITS paralogs in plant evolutionary studies is very risky because it can distort the phylogenetic signal (Mayol and Rosselló, 2001; Ma and Zhou, 2005; Feliner and Rosselló, 2007). To avoid incorporating ITS paralogs, PCR products need to be cloned into a vector for sequencing, which is time consuming and costly. Therefore, routine applicability of the ITS for barcoding is difficult.

Even single copy nuclear genes or a small batch of nuclear sequences may be not ideal barcoding makers for precise species identification in oaks, due to limited numbers of informative SNPs (Oh and Manos, 2008; Hubert et al., 2014). Recently, high-throughput markers, such as RAD-seq (Hipp et al., 2014; Cavender-Bares et al., 2015; Deng et al., 2017a,c) have demonstrated their power to successfully resolve the oak phylogeny with rich species and complex evolutionary history. Genome skimming also has great advantages for extending the plant barcode (Li et al., 2015; Coissac et al., 2016; Hollingsworth et al., 2016), allowing for the near-complete assembly of high-copy plastids, mitochondria and ribosomal DNA and possibly a fragmented nuclear genome assembly (Hollingsworth et al., 2016). These advanced technologies offer a promising future for DNA barcoding in oaks and probably other taxonomical difficult groups.

### Performance of Different Barcoding Analysis Methods

The different performances of the four methods may reflect the analytical theories implemented in each method. BLAST had the best performance, providing the highest species resolution for all single markers and combinations. BLAST calculates the similarity of sequences, and assume that individuals from a species will be more similar to those from other species (van Velzen et al., 2012). This method also yields higher identification rates in other barcoding studies in *Quercus, Curcuma, Rhododendron*, and bryophytes (Hassel et al., 2013; Chen et al., 2015; Yan et al., 2015; Yang et al., 2017). The distance-based method (ABGD) automatically finds the distance where the barcode gap is located to partition the data set into candidate species, and can be used even when the intra- and inter-species distances overlap (Puillandre et al., 2012). This explained though intra- and inter-species distances overlap in our dataset, which adds difficulty to discriminate species. Nevertheless, ABGD still provided a relatively high species discrimination rate. Conversely, the tree-based methods and diagnostic methods generate low species identification rate. The tree-based approach is depended on monophyletic status of species. However, we only found few species were resolved as monophyletic clades and successfully identified. The poor resolution of tree-based method is in concordance with other studies (Meier et al., 2006; Little and Stevenson, 2007; Virgilio et al., 2010; Little, 2011). It is worth noting that BLOG has been reported to generate the highest sequence identification success in oaks and other plants (van Velzen et al., 2012; Weitschek et al., 2013; Yang et al., 2017). Actually, in our study BLOG also provides the highest individual identification success (36.23%), but it demonstrated the lowest species identification success, which requires correct identification of all individuals of a species in both training and test sequences. BLOG identifies potential diagnostic nucleotide positions from training sequences and assigns species using logic formulas based on the species-specific (diagnostic) codes (Weitschek et al., 2013). However, the diagnostic codes based on training sequences may not always cover the total variance of all sequences. Meanwhile, the total sequences are divided into training and test datasets in BLOG, as a result there are fewer samples (only one or two) in each dataset. Fewer samples may increase the discrimination rate and lead to a superficially high success rate. Hence, the high success rate produced by BLOG should be checked carefully, whether a species is correctly identified in both training and test dataset.

### The Factors That Might Contributed to the Low Resolution of *cp*DNA in East Asian Evergreen Oaks

#### Conservation of the Chloroplast Genome

The poor species resolution of the four universal markers might be caused by multiple factors. Low mutation rates of chloroplast genome might lead to the poor resolution among different species. Actually, *cp*DNA has a low mutation rate, and much lower comparing to *nr*DNA (Lynch, 1997; Drouin et al., 2008). In theory, taxa with shorter generation time is likely to accumulate more mutations than long-lived species (Martin and Palumbi, 1993; Gaut et al., 1996). For woody plants with long reproductive cycle, their *cp*DNA mutation rates are much lower than the herbaceous species (Gaut et al., 1996). Generally, oaks have long generation time and oak seedlings need 3–45 years to produce their first acorns (Ducousso et al., 1993; Guyette et al., 2004; Kormanik et al., 2004). Consistent with the theory, the *cp*DNA substitution rates of oaks (0.19–0.96 × 10^-9^ s/s/y) (Chen et al., 2012; Xu et al., 2015, 2016; Feng et al., unpublished) are very low, below the average values reported for non-coding regions in other angiosperm lineages (1.2–1.7 × 10^-9^ s/s/y) (Graur and Li, 2000). Similarly, the low mutation rate of chloroplast genome is also reported in other woody species (Qi et al., 2012; Guo et al., 2014). The low substitution rate of *cp*DNA of oaks resulted in fewer mutations between different individuals, which partly explains the low species resolution of *cp*DNA in oaks. However, the low resolution may also be due to selective sweeps in chloroplast genome and past genetic drift (Ellstrand and Elam, 1993; Muir and Filatov, 2007; Percy et al., 2014). Because of the lower effective population size compared to nuclear genome, chloroplast genome may be greatly affected by genetic drift, which reduces the genetic diversity (Birky, 1988; Ellstrand and Elam, 1993; Schaal et al., 1998). As the result, chloroplast genome demonstrates low genetic diversity.

#### Complicated *cp*DNA Evolutionary History Involved Introgression and Incomplete Lineage Sorting

Chloroplast is maternally inherited in oaks (Dumolin et al., 1995). As a result, *cp*DNA is easy to transfer during the hybridization process, especially with asymmetric gene flow in the parental populations. Therefore, the *cp*DNA phylogeny only reflects the maternal evolutionary history. Oaks are notorious for interspecific hybridization, especially among the species in the same section (Curtu et al., 2007; Neophytou et al., 2011b; Moran et al., 2012; Leroy et al., 2017; McVay et al., 2017). Natural hybridization in East Asian evergreen oaks has been revealed and well demonstrated among sympatric species. In section *Cyclobalanopsis*, introgression between sympatric species (*Q. sessilifolia* and *Q. acuta* distributed in Korea and Japan; *Q. austrochinchinensis* and *Q. kerrii* distributed in Southwest China) were fully revealed using morphological traits and molecular markers (Tamaki and Okada, 2014; Song et al., 2015; An et al., 2017). The morphological intermediates have been frequently reported in section *Ilex* (Huang et al., 1999; Neophytou et al., 2007) and several hybridization zones with a series of morphological transitions have been discovered according to our field observation, indicating frequent interspecies hybridization. Meanwhile, the inconsistent evolutionary patterns between *cp*DNA and *nr*DNA were commonly detected in regional phylogeographic and population genetic analysis of section *Ilex*, which also implied introgression occurs in the Mediterranean and East Asia (Neophytou et al., 2011a,b; Meng et al., 2017; Vitelli et al., 2017). We found that the evolutionary history of the chloroplast genome is net-like rather than dichotomous, which hinders the discriminatory ability to infer the species tree using these maternal markers, especially in a frequent-hybridized taxa (Hollingsworth et al., 2011; Coissac et al., 2016). Moreover, driven by the peculiar paleogeographical histories of the studied regions, plastid genome may reveal geographic differentiation or distribution range of the ancestral lineage instead of the true species phylogeny (Petit et al., 2002; Gugger and Cavender-Bares, 2013; Simeone et al., 2016; Pham et al., 2017; Vitelli et al., 2017). Eurasian evergreen oaks also have strong *cp*DNA genetic differentiation across different geographical regions instead of species taxonomy. As we found in section *Ilex*, individuals from Mediterranean-Himalaya, SW China and East China formed independent clusters, respectively. It is likely that active tectonic movements induced uplift of SE Himalaya fringes and adjacent region, which blocked the regional seed-mediated gene flow and resulted in the strong *cp*DNA geographic differentiation in these oaks.

Another explanation for haplotype sharing between species which located across long distance is the incomplete lineage sorting of the ancestral lineages (Piredda et al., 2011; Simeone et al., 2013). In addition, occasional introgression following the long-distance dispersal events might result in this pattern as well (Lumaret et al., 2002; Toumi and Lumaret, 2010). Therefore, the low *cp*DNA mutation rates, strong asymmetric interspecies gene flow and incomplete lineage sorting of ancestral polymorphism may have contributed to the low resolution of *cp*DNA for species delimitation for East Asian evergreen oaks.

### Different Genetic Diversity Patterns in Asian Evergreen Lineages

#### Why Is the *cp*DNA Phylogeny Non-monophyletic?

Within the subgenus *Cerris*, our data shows that section *Ilex* is closely related to both sections *Cyclobalanopsis* and *Cerris*. A similar unresolved complex phylogenic relationship within subgenus *Cerris* was also found in other studies using *cp*DNA markers (Manos et al., 1999; Simeone et al., 2016). The chloroplast capture events within a genus are mostly due to hybridization (Fehrer et al., 2007; Acosta and Premoli, 2010). However, the hybridization between the extant sections in oaks is extremely rare in the wild (Costello et al., 2011). The non-monophyly of section *Ilex* plastomes and its close relationship with sections *Cyclobalanopsis* and *Cerris* may reflect an ancient introgression or incomplete lineage sorting of the chloroplast genome in the ancestor lineages of subgenus *Cerris*. In the future, this issue should be subjected to the further study using high-throughput molecular marker (e.g., GBS, RAD-seq) to resolve the species phylogeny. In addition, it needs to recruit multiple species populations of the three sections to reveal the ancient and/or contemporary gene flow among the lineages.

#### Why the Genetic Diversity in Sections of *Quercus* Is Different?

Theoretically, the deciduous lineages which are usually fast growing species, may have higher genetic variation, because they have shorter sexual maturation time comparing to evergreen lineages (Zuidema et al., 2009). Generally, it takes at least five or more years for seedlings of evergreen oaks to produce first acorns. But for the deciduous species, this cycle only needs 2–3 years (Wang and Gao, 2005; Wang et al., 2011), such as in *Q. serrata, Q. fabri*, and *Q. aliena.* However, the genetic variation patterns in oak sections may not match this theory well, as our study revealed that sections *Ilex* and *Quercus* have higher genetic variation, and other sections have lower variation. This result is slightly different to previous reports that sections *Ilex, Lobatae* and *Quercus* were the most variable lineages, and section *Cerris* was the least variable (Simeone et al., 2016). Currently, it is too early to conclude the overall genetic diversity patterns of the genus *Quercus*, because of the uneven sampling of the main sections.

However, in the well-sampled Asian evergreen sections *Cyclobalanopsis* and *Ilex*, we found different patterns of genetic diversity. Trees of section *Ilex* grow at semi-arid habitats with slow biomass accumulation ratio and long sexual maturation cycle (Mediavilla and Escudero, 2003). All these biological traits of species of section *Ilex* tend to cause low *cp*DNA genetic diversity level. However, it was the most diverse lineage based on our study. Interestingly, section *Cyclobalanopsis* with high number of species and great morphological variation showed low genetic diversity. The different biogeographical histories and adaptabilities to the environment in the two sections may have driven their genetic diversity patterns. Section *Cyclobalanopsis* was an early derived (at the early Oligocene) section of subgenus *Cerris*, but their lineage diversification did not occur until the early Miocene and the highest diversification ratio achieved at the mid-late Miocene (Deng et al., 2017a). Instead, section *Ilex* was later derived at the early-middle Oligocene, but it shows a stepwise diversification pattern since the middle Oligocene onward (Deng et al., 2017c). Therefore, most species in section *Cyclobalanopsis* are probably younger than those in section *Ilex*. With longer evolutionary time, more *cp*DNA mutations may have accumulated, which may explain why the genetic diversity level of section *Ilex* is much higher than that of section *Cyclobalanopsis*. Moreover, section *Ilex* can grow in a wide geographic range with diversified habitats, aside from the prominent geographic barriers, the local adaptation to different environments may have also played a role in boosting the genetic diversity of section *Ilex* (Du et al., 2016; Feng et al., 2016; Meng et al., 2017; Feng et al., unpublished). In contrast, trees in section *Cyclobalanopsis* live mainly in warm and humid subtropical habitats (Huang et al., 1999; Deng et al., 2017a). As dominant trees, their large effective population size and the similar habitats may make them less influenced by topographic and climate changes. Shorter divergence time, long-term environment stability and strong gene flow among regional populations, may have altogether resulted in the low genetic diversity of the *cp*DNA in section *Cyclobalanopsis*.

## Conclusion and Perspective

This study reveals that the *cp*DNA markers have limited efficiency to identify the Asian evergreen oaks, but these *cp*DNA markers are still informative to infer the species placement to the main sections of *Quercus*. The different genetic diversity patterns between the two evergreen oak sections *Ilex* and *Cyclobalanopsis* may have been shaped by their spatio-temporal histories and different adaptabilities to environments. The different evolutionary pattern of oaks revealed by the chloroplast and nuclear genomes is most likely due to the historical introgression in the ancestral lineages and recent or on-going gene flow between the closely related species. All these factors reduce the efficiency of *cp*DNA as species level barcodes. Even single copy nuclear genes or a small batch of nuclear sequences may not be ideal barcoding makers for precise species identification in oaks, due to limited number of informative SNPs. The advanced technologies, such as high-throughput markers (e.g., RAD-seq and GBS) and genome skimming offer a promising future for DNA barcoding in oaks and other taxonomic difficult groups.

## Author Contributions

MD conceived and designed the experiments. YX, RL, and MY performed the experiments. MY and JS analyzed the data. MD was responsible for field collections and specimen identification. MY and MD wrote and revised the paper.

## Conflict of Interest Statement

The authors declare that the research was conducted in the absence of any commercial or financial relationships that could be construed as a potential conflict of interest.

## Footnotes

1 http://www.megasoftware.net/

2 http://tree.bio.ed.ac.uk/software/tracer/

## Abbreviations

*cp*DNA: chloroplast DNA
EBLF: evergreen broad-leaved forest
